# Increased age and male sex are independently associated with higher frequency of blood–cerebrospinal fluid barrier dysfunction using the albumin quotient

**DOI:** 10.1186/s12987-020-0173-2

**Published:** 2020-02-05

**Authors:** Massimiliano Castellazzi, Andrea Morotti, Carmine Tamborino, Francesca Alessi, Silvy Pilotto, Eleonora Baldi, Luisa M. Caniatti, Alessandro Trentini, Ilaria Casetta, Enrico Granieri, Maura Pugliatti, Enrico Fainardi, Tiziana Bellini

**Affiliations:** 1 Department of Biomedical and Specialist Surgical Sciences, University of Ferrara, Via Aldo Moro 8, Settore 1C3, 44124 Ferrara, Italy; 2 Interdepartmental Research Center for the Study of Multiple Sclerosis and Inflammatory and Degenerative Diseases of the Nervous System, University of Ferrara, Ferrara, Italy; 3 Stroke Unit, IRCCS Mondino Foundation, Pavia, Italy; 4 Neurology Unit, Azienda Ulss 3 Serenissima, Mestre, Italy; 5 School of Medicine, University of Ferrara, Ferrara, Italy; 6 Department of Neuroscience and Rehabilitation, Azienda Ospedaliero-Universitaria di Ferrara, Ferrara, Italy; 7 Department of Experimental and Clinical Biomedical Sciences, University of Florence, Florence, Italy; 8 University Center for Studies on Gender Medicine, University of Ferrara, Ferrara, Italy

**Keywords:** Quotient of albumin (QAlb), Sex, Age, Cerebrospinal fluid (CSF) analysis, Blood–cerebrospinal fluid barrier (B-CSF-B)

## Abstract

**Background:**

The cerebrospinal fluid (CSF)/serum quotient of albumin (QAlb) is the most used biomarker for the evaluation of blood–cerebrospinal fluid barrier (B-CSF-B) permeability. For years QAlb was considered only as an age-related parameter but recently it has also been associated to sex. The aim of the present study was to explore the impact of sex in the determination of B-CSF-B dysfunction.

**Methods:**

The analysis was retrospectively conducted on subjects consecutively admitted to the neurological ward. CSF and serum albumin levels were measured by immunonephelometry and pathological QAlb thresholds were considered: 6.5 under 40 years, 8.0 in the age 40–60 and 9.0 over 60 years.

**Results:**

1209 subjects were included in the study. 718 females and 491 males (age: 15–88 years): 24.6% of patients had a diagnosis of multiple sclerosis, 23.2% suffered from other inflammatory neurological diseases, 24.6% were affected by non-inflammatory neurological diseases, and for 27.6% of patients the final neurological diagnosis could not be traced. Dysfunctional B-CSF-B was detected more frequently (44 vs. 20.1%, p < 0.0001) and median QAlb value were higher (7.18 vs. 4.87, p < 0.0001) in males than in females in the overall study population and in all disease subgroups. QAlb and age were positively correlated both in female (p < 0.0001) and male (p < 0.0001) patients, however the slopes of the two regression lines were not significantly different (p = 0.7149), while the difference between the elevations was extremely significant (p < 0.0001) with a gap of 2.2 units between the two sexes. Finally, in a multivariable linear regression analysis increased age and male sex were independently associated with higher QAlb in the overall study population (both p < 0.001) and after stratification by age and disease group.

**Conclusions:**

Accordingly, identification and validation of sex-targeted QAlb thresholds should be considered as a novel tool in an effort to achieve more precision in the medical approach.

## Background

Brain homeostasis is maintained by means of two different barriers: the blood–cerebrospinal fluid (CSF) barrier (B-CSF-B) formed by the choroid plexus epithelial cell, and the blood–brain barrier (BBB) formed by the cerebral blood vessel endothelium [1, 2]. Since both barriers are in chemical–physical equilibrium with each other and functionally overlap, the term ‘BBB’ is most commonly used simply to refer to the entire functional system separating blood from CSF and nervous tissue [3]. In clinical routine, the CSF/serum quotient of albumin—the most concentrated protein in the CSF-, namely QAlb, is the most used and reliable biomarker to assess B-CSF-B permeability [4, 5]. Albumin is produced in the liver and is not catabolised within the central nervous system (CNS), and it is generally thought that all albumin measured in CSF is of plasma derivation [6], although there is some evidence that glial cells can produce albumin [7].

Within threshold values QAlb indicates a “normal” B-CSF-B permeability, while values beyond the threshold reflect B-CSF-B dysfunction [8]. The use of QAlb is preferable to the simple measurement of CSF total proteins [9]. An isolated abnormal elevation of QAlb with no other CSF pathological sign has been described in acute and chronic inflammatory demyelinating polyneuropathy and normal pressure hydrocephalus, but has also been seen in patients with no neurological disease [10]. QAlb threshold values increase with age [8, 11] indicating an age-related progressive loss of B-CSF-B integrity [12], reduced CSF flow rate [13] and a decreased CSF turnover [14]. While the dependence between QAlb and age is clear [15], evidence of sex-related differences is less explored [16].

QAlb values were recently reported to be higher in men than women regardless of age in a large population of patients with unspecified disorders and in healthy volunteers [17]. The implication of QAlb sex-related differences in clinical practice was, however, not addressed.

Owing to sex-related differences in brain development, structure, and neurotransmission [18], exploring sexual dimorphisms in the brain may be key to understanding the role of sex in predisposing to neurological diseases [19].

Our aim was to investigate whether sex has an impact on B-CSF-B permeability by analysing QAlb in a cohort of neurological patients.

## Methods

### Study design

Clinical and laboratory anonymized data were retrospectively collected from patients hospitalized from 2000 to 2018 in the ‘S. Anna’ University Hospital (Azienda Ospedaliero-Universitaria S. Anna), in Ferrara, northern Italy. The study was approved by the local Committee for Medical Ethics in Research, “Comitato Etico di Area Vasta Emilia Centro della Regione Emilia-Romagna” (Prot. N. 770/2018/Oss/AOUFe, dated 12/12/2018) and written informed consent was obtained.

### Patients and methods

All patients included in the study had undergone lumbar puncture for diagnostic purposes. Blood and CSF samples had been withdrawn at the same time. CSF and serum samples were analysed at room temperature immediately after centrifugation or stored in aliquots at − 80 °C until assay. All analyses were performed on the 2nd–4th ml of CSF after lumbar puncture. For every patient albumin levels were measured as part of the diagnostic work-up in cell-free CSF and paired serum samples by immunochemical nephelometry with the Beckman Array Protein System or IMMAGE 800 Immunochemistry System (Beckman Instruments, Fullerton, CA, USA) according to the procedure of Salden [20]. Albumin quotient was calculated to disclose B-CSF-B dysfunction according to the formula: QAlb = [Alb]CSF/[Alb]serum × 1000. Normal QAlb values were considered as < 6.5 for patients aged 15–40 years, < 8.0 for patients aged 41–60 years and < 9.0 for patients over 60 years [8, 9, 21]. Accordingly, QAlb was considered as pathological for values greater than or equal to the reported thresholds.

Exclusion criteria were (i) age less than 16 years (ii) lack of demographic data (sex and/or age) and (iii) the presence of xanthochromia and/or high levels of CSF red blood cells (> 2000/µl) as suggestive of traumatic lumbar puncture or subarachnoid haemorrhage.

These subjects were subsequently diagnosed with multiple sclerosis (MS), other inflammatory neurological diseases (OIND), including inflammatory diseases of both central and peripheral nervous systems, non-inflammatory neurological diseases (NIND) and patients with untraceable definite neurological diagnosis (UNK). For all subjects, sex, age, and QAlb values were collected.

### Statistical analysis

Continuous variables with non-normal distribution at the Kolmogorov–Smirnov test were reported as median (interquartile range, IQR) and comparisons were made with Mann–Whitney test. Categorical variables were reported as count (percentage) and Chi square test used for comparison. Correlations between age and QAlb were investigated with Spearman test. In regression analysis F-test was used to compare the fits of linear models. Independent predictors of Log-transformed QAlb were explored using multivariable linear regression, and age and sex were used as reciprocal covariates in the model. Two tailed p-values < 0.05 were considered statistically significant. The Statistical Package for the Social Sciences (SPSS^®^) version 21.0 for Windows, OSX (SPSS Inc., IBM^®^, Somers, New York, USA) and Prism^®^ 8 (GraphPad Software Inc.) were used for the statistical analysis.

## Results

### Patients characteristics

The study was conducted on 1209 individuals, 718 women and 491 men, aged between 15 and 88 years: 297 (24.6%) patients had a diagnosis of MS, 281 (23.2%) suffered from OIND, 297 (24.6%) were affected by NIND, and for 334 (27.6%) the definite neurological diagnosis could not be traced (UNK). The main clinical-demographic features of the study population are reported in Table 1.

**Table 1:** Clinical and demographic characteristics of the study population

	Female	Male	p
All patients, n (%)	718 (59.4)	491 (40.6)	
Age, years: median (IQR)	44 (33–58)	48 (35–65)	0.0002
MS, n (%)	204 (68.7)	93 (31.3)	
Age, years: median (IQR)	37.0 (30.0–45.0)	35.0 (29.0–45.0)	0.5289
Relapsing–remitting: n	163	72	
Secondary progressive: n	23	11	
Primary progressive: n	18	10	
OIND, n (%)	132 (47.0)	149 (53.0)	
Age, years: median (IQR)	52.5 (39.0–63.6)	52.0 (38.5–69.0)	0.5070
Diagnosis (n)	Infectious diseases (17) Autoimmune neurological diseases (46) Paraneoplastic CNS neurological syndromes (4) Aseptic or bacterial meningitis; aseptic encephalitis or myelitis (16) Inflammatory demyelinating neuropathies (40). Inflammatory neuritis (9)	Infectious diseases (15) Autoimmune neurological diseases (26) Paraneoplastic CNS neurological syndromes (10) Aseptic or bacterial meningitis; aseptic encephalitis or myelitis (26) Inflammatory demyelinating neuropathies (59) Inflammatory neuritis (13)	
NIND, n (%)	146 (49.2)	151 (50.8)	
Age, years: median (IQR)	52.0 (38.8–68.0)	53.0 (37.0–66.0)	0.7482
Diagnosis (n)	CNS expansions (14) Vascular diseases (50) Neurodegenerative diseases (20) Hereditary/metabolic encephalopathies (4) Unconsciousness (18) Non-inflammatory PNS involvement (16) CSF flow abnormalities (5) Dementia and Parkinson syndromes (19)	CNS expansions (13) Vascular diseases (51) Neurodegenerative diseases (27) Hereditary/metabolic encephalopathies (2) Unconsciousness (10) Non-inflammatory PNS involvement (27) CSF flow abnormalities (2) Dementia and Parkinson syndromes (19)	
UNK, n (%)	236 (70.7)	98 (29.3)	
Age, years: median (IQR)	42.4 (31.0–58.0)	51.0 (40.8–66.0)	0.0002

Age distributions were compared with Mann–Whitney u-test

*CNS* central nervous system, *CSF* cerebrospinal fluid, *MS* multiple sclerosis, *NIND* non-inflammatory neurological diseases, *OIND* other inflammatory neurological diseases, *PNS* peripheral nervous system, *UNK* unknown-neurological diagnosis

The women:men ratio was 1.46:1 in the overall study population, 2.19:1 in MS, 0.89:1 in OIND, 0.97:1 in NIND and 2.41:1 in UNK group. In the overall study population age at admission was higher in men (48.0 years) than in women (44.0 years) and in the UNK subgroup it was 51.0 years versus 42.4 years, respectively. No further significant difference in age distribution was observed across groups.

### Distribution of the B-CSF-B dysfunction in the study population grouped by age and diagnosis

In our overall study population, based on QAlb, men were reported with a B-CSF-B dysfunction more frequently than women (44.0% vs. 20.1%). After stratification by age, 40.8% men vs. 11.8% women under 40 years of age were reported with B-CSF-B dysfunction, 44.3% vs. 23.4% between 41 and 60 years, and 47.1% vs. 33.3% over 60 years (Table 2). This sex-difference was confirmed in all the disease subgroups: 24.7% vs. 7.35% in MS men and women, respectively, 59.7% vs. 45.5% in patients with OIND, 33.1% vs. 15.8% in NIND and 55.1% vs. 22.0% in the UNK group.

**Table 2:** Frequency of diagnoses of blood–CSF barrier (B–CSF–B) dysfunction in the study population by sex, age and specific disease status

	Female	Male	p
All: n (%)	718 (59.4)	491 (40.6)	
Altered B-CSF-B: n (%)	150 (20.1)	216 (44.0)	< 0.0001
Age 16–40 years: n (%)	37 (11.8)	66 (40.8)	< 0.0001
Age 41–60 years: n (%)	60 (23.4)	78 (44.3)	< 0.0001
Age > 60 years: n (%)	53 (33.3)	72 (47.1)	0.0134
MS: n (%)	204 (68.7)	93 (31.3)	
Altered B-CSF-B: n (%)	15 (7.35)	23 (24.7)	< 0.0001
OIND: n (%)	132 (47.0)	149 (53.0)	
Altered B-CSF-B: n (%)	60 (45.5)	89 (59.7)	0.0167
NIND: n (%)	146 (49.2)	151 (50.8)	
Altered B-CSF-B: n (%)	23 (15.8)	50 (33.1)	0.0005
UNK: n (%)	236 (70.7)	98 (29.3)	
Altered B–CSF–B: n (%)	52 (22.0)	54 (55.1)	< 0.0001

B-CSF-B dysfunction was defined using the cerebrospinal fluid (CSF)/serum albumin quotient (QAlb) with the following upper reference limits: 6.5 for patients aged 16–40 years, 8.0 for patients aged 41–60 years and 9.0 for patients over 60 years. Chi square test was used for all comparisons

*ALL* patient groups analysed as a whole, *MS* multiple sclerosis, *NIND* non-inflammatory neurological diseases, *OIND* other inflammatory neurological diseases, *UNK* unknown-neurological diagnosis

### Medians of QAlb values in the study population and prevalence of sex across QAlb quartiles

Median QAlb values were significantly higher in the male than in the female overall study population (7.18 vs. 4.87), and in all age subgroups: 5.74 vs. 4.19 in the under 40 s, 7.33 vs. 5.03 in the group aged between 41 and 60 years, and 8.87 vs. 6.52 in the over 60 s (Table 3). This sex-difference was also found in all the disease subgroups: 5.22 vs. 4.10 in MS, 9.22 vs. 6.68 in OIND, 6.34 vs. 5.09 in NIND and 8.48 vs. 4.89 in UNK (Table 3).

**Table 3:** Albumin quotient (QAlb) distribution in the study population by sex, age and specific disease status

	Females	Males	p
ALL, n (%)	718 (59.4)	491 (40.6)	
QAlb: median (IQR)	4.87 (3.69–6.88)	7.18 (4.91–11.30)	< 0.0001
Age 16–40 years: median (IQR)	4.19 (3.28–5.41)	5.74 (4.40–9.20)	< 0.0001
Age 41–60 years: median (IQR)	5.03 (3.88–7.83)	7.33 (4.87–11.26)	< 0.0001
Age > 60 years: median (IQR)	6.52 (4.93–10.96)	8.87 (5.82–15.43)	0.0008
MS, n (%)	204 (68.7)	93 (31.3)	
QAlb: median (IQR)	4.10 (3.27–5.36)	5.22 (4.15–7.61)	< 0.0001
OIND, n (%)	132 (47.0)	149 (53.0)	
QAlb: median (IQR)	6.68 (4.54–13.1)	9.22 (5.65–15.00)	0.0172
NIND, n (%)	146 (49.2)	151 (50.8)	
QAlb: median (IQR)	5.09 (3.87–6.58)	6.34 (4.64–9.42)	< 0.0001
UNK, n (%)	236 (70.7)	98 (29.3)	
QAlb: median (IQR)	4.89 (3.70–7.38)	8.48 (6.2–12.4)	< 0.0001

Mann–Whitney u-test was used for all comparisons

*ALL* patient groups analysed as a whole, *MS* multiple sclerosis, *NIND* non-inflammatory neurological diseases, *OIND* other inflammatory neurological diseases, *UNK* unknown-neurological diagnosis

QAlb values were stratified by quartiles (Table 4). Prevalence of the female sex was higher in the lower quartiles (Chi square test: p < 0.0001) in the overall study population and across all disease subgroups (Chi square test: MS, p < 0.001; OIND, p = 0.0309; NIND, p = 0.0002; UNK, p < 0.0001), also after stratification by age (Chi square test: age 16-40 years, p < 0.0001; age 41–60 years, p < 0.0001; age > 60 years, p = 0.0071).

**Table 4:** Prevalence of sex across albumin quotient (QAlb) quartiles

	Q1 (QAlb < 4.06)	Q2 (4.06 < QAlb < 5.57)	Q3 (5.58 < QAlb < 8.97)	Q4 (QAlb > 8.98)	p
All (n = 1209)					
Female: n (%)	244 (81.3)	194 (63.6)	158 (52.3)	122 (40.4)	<0.0001
Male: n (%)	56 (18.7)	11 (36.4)	144 (47.7)	180 (59.6)	
MS (n = 297)					
Female: n (%)	101 (82.8)	58 (67.4)	35 (53.8)	10 (41.7)	<0.0001
Male: n (%)	21 (17.2)	28 (32.6)	30 (46.2)	14 (58.3)	
OIND (n = 281)					
Female: n (%)	23 (67.7)	25 (51.0)	31 (47.7)	53 (39.8)	0.0309
Male: n (%)	11 (32.4)	24 (49.0)	34 (52.3)	80 (60.2)	
NIND (n = 297)					
Female: n (%)	43 (71.7)	47 (50.0)	37 (44.6)	19 (31.7)	0.0002
Male: n (%)	17 (28.3)	47 (50.0)	46 (55.4)	41 (68.3)	
UNK (n = 334)					
Female: n (%)	77 (91.7)	64 (84.2)	55 (61.8)	40 (47.1)	<0.0001
Male: n (%)	7 (8.3)	12 (15.8)	34 (38.2)	45 (52.9)	
Age 16–40 (n = 465)					
Female: n (%)	139 (84.2)	92(64.3)	58 (57.4)	14 (25.0)	<0.0001
Male: n (%)	26 (15.8)	51 (35.7)	43 (42.6)	42 (75.0)	
Age 41–60 (n = 435)					
Female: n (%)	83 (78.3)	63 (64.9)	57 (51.4)	53 (43.8)	<0.0001
Male: n (%)	23 (21.7)	34 (35.1)	54 (48.6)	68 (56.2)	
Age > 60 (n = 309)					
Female: n (%)	22 (75.9)	39 (60.0)	43 (47.8)	55 (44.0)	0.0071
Male: n (%)	7 (24.1)	26 (40.0)	47 (52.2)	70 (56.0)	

Chi square test was used for all comparisons

*ALL* patient groups analysed as a whole, *MS* multiple sclerosis, *NIND* non-inflammatory neurological diseases, *OIND* other inflammatory neurological diseases, *UNK* unknown-neurological diagnosis

### Associations between age, sex and QAlb

**Fig. 1:**
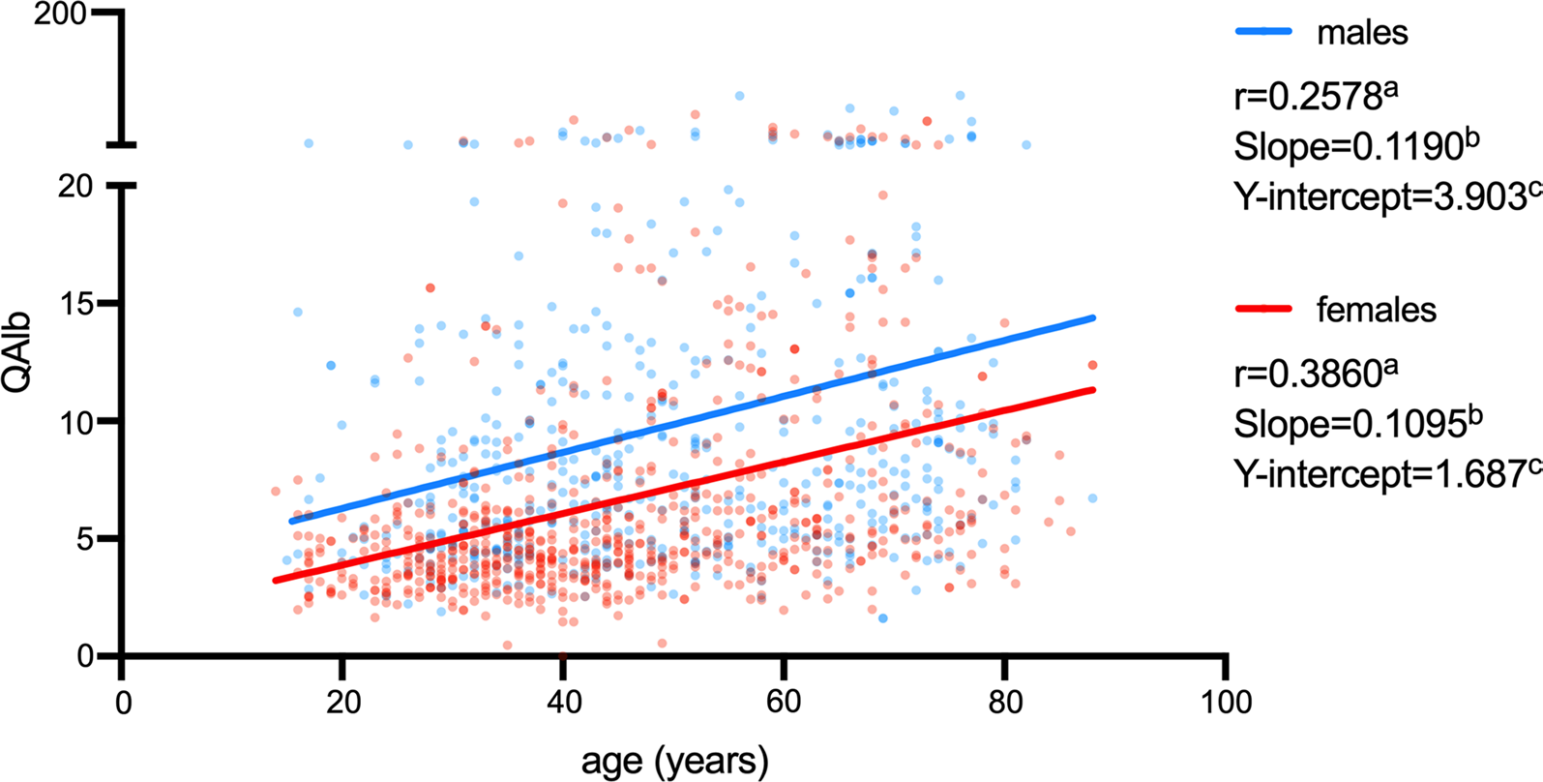
Linear regression analysis of albumin quotient (QAlb) and age in male and female patients. ^a^QAlb and age were positively correlated in male (Spearman, p < 0.0001) and female (Spearman, p < 0.0001) patients. ^b^The slopes of the two regression lines were not significantly different (F test, p = 0.7149). ^c^The difference between the elevations was significant (F test, p < 0.0001)

In the entire study population, QAlb positively correlated with age both in women (r = 0.383, p < 0.0001) and in men (r = 0.2578, p < 0.0001) (Fig. 1). The slopes of the sex-specific regression lines were not significantly different (F = 0.1335, p = 0.7149), while the difference between the elevations (1.687 in women vs. 3.903 in men) was very significant (F = 37.65, p < 0.0001).In multivariable linear regression analysis, both increasing age and sex (M) were independently associated with higher QAlb in the whole population and in MS, OIND, NIND and UNK subgroups (p < 0.001 for all subgroups) (Table 5).

**Table 5:** Predictors of albumin quotient (QAlb)

	B (SE)	p
ALL (n = 1209)		
Age	0.31 (0.00)	< 0.001
Sex, M	0.25 (0.02)	< 0.001
MS (n = 297)		
Age	0.24 (0.01)	< 0.001
Sex, M	0.29 (0.02)	< 0.001
OID (n = 281)		
Age	0.22 (0.00)	< 0.001
Sex, M	0.12 (0.04)	< 0.001
NID (n = 297)		
Age	0.24 (0.00)	< 0.001
Sex, M	0.28 (0.03)	< 0.001
UNK (n = 334)		
Age	0.29 (0.00)	< 0.001
Sex, M	0.32 (0.03)	< 0.001

log (QAlb) in multivariate regression analysis

*ALL* patient groups analysed as a whole, *MS* multiple sclerosis, *NIND* non-inflammatory neurological diseases, *OIND* other inflammatory neurological diseases, *UNK* unknown-neurological diagnosis

### Correlations between age and albumin levels

**Fig. 2:**
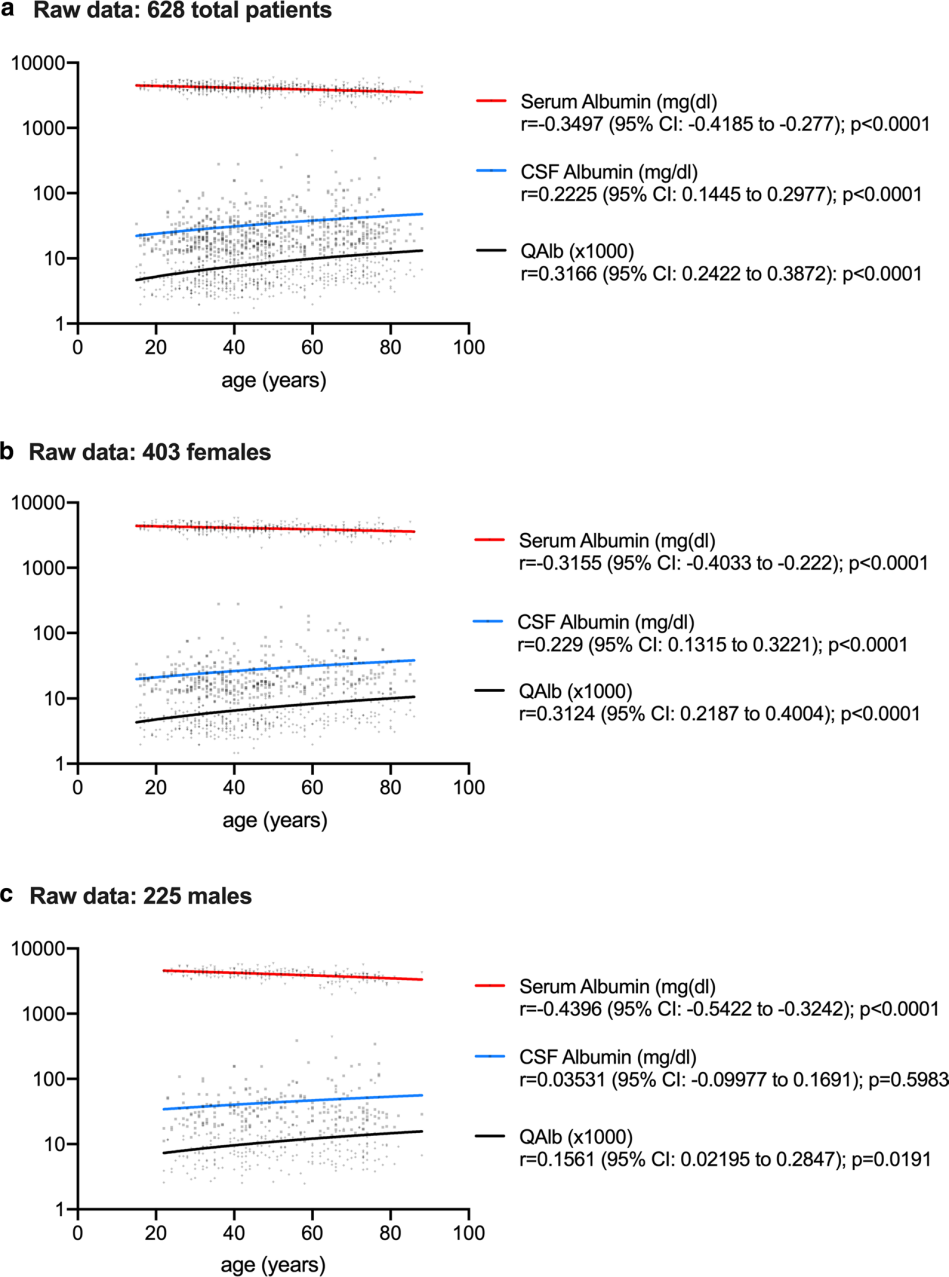
Correlations between age and albumin levels in cerebrospinal fluid (CSF) and serum. Serum albumin levels decreased with age in all patients analysed as a whole (Spearman: p < 0.0001) and grouped by sex (Spearman: p < 0.0001 for woman and p < 0.0001 for men). CSF albumin levels increased with age in the entire population (Spearman: p < 0.0001) and in the female subgroup (Spearman: p < 0.0001). The CSF/serum albumin ratio (QAlb) increased with age in all patients analysed as a whole (Spearman: p < 0.0001) and grouped by sex (Spearman: p < 0.0001 in women and p = 0.0191 in men).* CI* confidence interval

Raw CSF and serum data were available for 628 patients: 108 MS (75 females, 33 males); 92 OIND (42 females, 50 males); 94 NIND (50 females, 44 males); 334 UNK (236 females, 98 males). As reported in Fig. 2, serum albumin concentrations were negatively correlated to age in all patients analysed as a whole (r = − 0.3497, p < 0.0001) and in female and male subgroups (r = − 0.3155, p < 0.0001 and r = − 0.4396, p < 0.0001, respectively). CSF albumin levels were positively correlated to age in the entire population (r = 0.2225, p < 0.0001) and in the female subgroup (r = 0.229, p < 0.0001). QAlb was positively correlated to age in all patients analysed as a whole (r = 0.3166, p < 0.0001) and grouped by sex (r = 0.3124, p < 0.0001 in females; r = 0.1561, p = 0.0191 in males).

### Correlations between QAlb and disability in multiple sclerosis patients

At the time of sample collection disease severity was scored using Kurtzke’s Expanded Disability Status Scale (EDSS) [22] in 286 MS patients (199 women and 87 men). EDSS values were similar (Mann–Whitney: p = 0.4343) between women (median and interquartile range (IQR): 2.5, 1.5–3.5) and men (median and IQR: 3.0, 1.5–3.5). QAlb values were positively correlated to the EDSS in the MS group analysed as a whole (Spearman: r = 0.1535, 95% CI 0.03481 to 0.2680; p = 0.0093) and in the females subgroup (Spearman: r = 0.1789, 95% CI 0.03666 to 0.3140; p = 0.0115), data not shown.

## Discussion

In our study we have shown that both older age and male sex are independently associated with higher QAlb in a large population of neurological patients. Because QAlb reflects B-CSF-B dysfunction [4, 5], this condition is therefore more frequently assigned to men than to women, irrespective of age and specific disease status.

Our findings are in line with those from a study by Parrado-Fernández et al. which was recently conducted in a population of over 20,000 patients with unspecified diseases as well as in control subjects [17]. They observed significantly higher QAlb in men than in women and irrespective of age, but the implications of such a sex discrepancy on B-CSF-B dysfunction were not discussed (e.g., in terms of percentage of patients positive for abnormal QAlb value).

Since it was introduced in 1977 [8], QAlb has been considered the best marker of B-CSF-B permeability dysfunction, the “B-CSF-B dysfunction”, by different expert panels [4, 9, 23, 24].

QAlb is reported to be normal or only rarely increased in MS [9]. In the present work, we observed that nearly 25% of male, compared to less than 10% of female, MS patients reported levels of QAlb which may indicate B-CSF-B dysfunction. Moreover, here we report that in MS patients, and particularly in females, an increase in disease severity seems to be associated to an altered B-CSF-B permeability. Now, we can only speculate that this increase could be the consequence of a modified posture and/or a reduced physical activity due to the disease progression [25, 26].

Elevated QAlb values have been reported above all in OIND and also, although less frequently, in NIND and in patients without neurological disorders [10, 27]. Accordingly, in our study population, for almost 60% of OIND and 33% of NIND male patients, an altered B-CSF-B could ultimately be reported, regardless of the inflammatory nature of the disease.

As has also been observed in other studies [28], in our population of neurological patients, QAlb increased with age while serum albumin levels decreased regardless of sex, whereas CSF levels remained almost stable in men and increased significantly in women. These observations confirm that B-CSF-B permeability to albumin changes in relation to age in the two sexes, albeit in a different way.

Sex-specific differences in QAlb values may be secondary to a number of mechanisms. The female hormone, 17β-estradiol [29], may drive a different expression of the enzymes involved in the BBB breakdown [30, 31], ultimately leading to a protective role on BBB [32]. Interestingly, because QAlb sex-specific values do not change in puberty or menopause, the role of a genetic predisposition linked to sex chromosomes together with that of hormones themselves should be considered [17, 33].

Also, a difference in the CSF flow rate between sexes may explain the sex-specific differences in QAlb. For decades QAlb has been regarded as increasing with age [9, 13, 34], in relation to the reduced speed of CSF in the elderly [35], and an increase in CSF albumin concentration has also been associated to the reduced CSF turnover occuring in the aging process [16, 36].

Other factors such as the use of oral contraception in females [37] or posture and physical activity [25] have been reported to influence the albumin CSF/serum ratio, however, to date a widely recognized agreement on the actual role of these potentially confounding factors is still lacking.

From another perspective, the different distribution of QAlb defined B-CSF-B dysfunction by sex indeed opens up a discussion on the relevant potential implications for clinical practice. If this difference is genetically determined [17, 33], we believe that QAlb threshold values should be normalized by sex and age in order not to overestimate B-CSF-B alteration in men, or, on the contrary, underestimate it in women. In the present work, QAlb values appear to be 2.2 units higher in men than in women irrespective of age and neurological pathology. This coefficient might be considered for future establishment of QAlb thresholds.

Even in the absence of B-CSF-B alteration, however, the sex-specific difference of QAlb may still reflect corresponding differences in B-CSF-B permeability and/or CSF flow/turnover also in normal subjects. In this perspective, differential drug delivery to the CNS should be hypothesized, and sex-specific pharmacological strategies may be required to ensure the most effective drug concentration crossing the BBB.

Finally, it is worth reporting that it has recently been shown that glial cells in the CNS can produce albumin [7, 38–40], but how much this contributes to the total CSF albumin concentration is still being debated. QAlb, therefore, still remains the only validated laboratory tool for assessing B-CSF-B permeability.

Our study has some limitations. The analysis did not include a healthy donor population so a comparison between neurological patients and a healthy population could not be made. Thus, it is possible that the male/female and age differences might be due to altered responses to disease. However, we did include groups with different neurological pathologies, i.e., MS, OIND and NIND, which ultimately showed similar QAlb distribution, and were in line with existing evidence on healthy subjects reported elsewhere [17]. Furthermore, we acknowledge that a specific neurological diagnosis was not available for ca 25% of the study population. However, these were patients who had been admitted to the Unit of Neurology and who could have been assigned to any of the three groups, across which no differential pattern of QAlb distribution by sex and age was observed.

## Conclusions

Our study reveals that, regardless of age, male neurological patients show B-CSF-B dysfunction more frequently than females, based on QAlb values. This evidence opens up discussion on whether the current QAlb threshold needs to be revised e.g., by introducing a corrective factor, in consideration of sex-genetically background differences, or whether B-CSF-B permeability truly differs between sexes, making men more prone to B-CSF-B dysfunction than women. In any case, future studies are required to evaluate whether the gap of 2.2 units we found between male and female neurological patients might be applied as a sex-related corrective factor to the thresholds for the evaluation of B-CSF-B permeability through the QAlb.

In an era of cost-effective personalised medicine, these considerations could have practical implications on sex-specific prognosis of neurological disorders as well as on the choice of the best strategy for drug delivery into the CNS.

## Data Availability

The datasets used and analysed during the current study are available from the corresponding author on reasonable request.

## Abbreviations

BBB: Blood–brain barrier
B-CSF-B: Blood–cerebrospinal fluid-barrier
CI: Confidence interval
CNS: Central nervous system
CSF: Cerebrospinal fluid
IQR: Interquartile range
MS: Multiple sclerosis
NIND: Non inflammatory neurological diseases
OIND: Other inflammatory neurological diseases
QAlb: Quotient of albumin
UNK: Patients with untraceable definite neurological diagnosis
